# Cauda Equina Syndrome Without Perineal Sensory Changes or Lower Extremity Neurological Deficits Following Postoperative Spinal Epidural Hematoma: A Case Report and Literature Review

**DOI:** 10.1111/os.14343

**Published:** 2025-01-07

**Authors:** Guanyi Liu, Qing Li, Hongfeng Ruan, Bingke Zhu, Weihu Ma, Yong Hu

**Affiliations:** ^1^ Department of Orthopedics Ningbo No. 6 Hospital Ningbo Zhejiang China; ^2^ Department of Endocrinology Ningbo Yinzhou No. 2 Hospital Ningbo Zhejiang China; ^3^ Institute of Orthopedics and Traumatology The First Affiliated Hospital of Zhejiang Chinese Medical University (Zhejiang Provincial Hospital of Traditional Chinese Medicine) Zhejiang Hangzhou China

**Keywords:** cauda equina syndrome, lumbar microdiscectomy, neurological recovery, spinal epidural hematoma, urinary retention

## Abstract

**Background:**

Postoperative spinal epidural hematoma (SEH) is a rare but serious complication following lumbar surgery, with cauda equina syndrome (CES) being one of its most devastating outcomes. While CES typically presents with a combination of bladder and/or bowel dysfunction, diminished sensation in the saddle area, and motor or sensory changes in the lower limbs, atypical cases with isolated urinary symptoms are less recognized and pose significant diagnostic challenges.

**Case Presentation:**

We report the case of a 46‐year‐old male who developed CES following lumbar microdiscectomy, presenting solely with urinary retention, without the classic signs of lower limb weakness or perineal sensory loss. Initial symptoms were attributed to postoperative urinary issues, delaying the diagnosis of CES. On postoperative day 7, magnetic resonance imaging (MRI) revealed SEH, and emergency hematoma evacuation was performed. Despite the delayed intervention, the patient made a full neurological recovery, with bladder and bowel functions restored by 3 months postoperatively.

**Conclusion:**

This case highlights the importance of recognizing CES in patients with isolated urinary dysfunction after lumbar surgery, even when typical neurological symptoms such as lower limb weakness or perineal sensory loss are absent. Early detection and prompt surgical intervention are critical, as delayed diagnosis may result in permanent neurological deficits. Moreover, this case underscores the need for vigilant postoperative monitoring, especially of urinary function, as isolated urinary symptoms may signal early CES. Maintaining a high index of suspicion for CES, even in atypical presentations, can facilitate timely diagnosis and improve patient outcomes. Furthermore, this case highlights the need for continued research into the prevention of SEH and the development of more robust diagnostic criteria for CES in postoperative patients. Future studies should focus on developing more comprehensive guidelines for monitoring postoperative patients, especially regarding urinary function, to aid in the early detection of CES.

## Graphical abstract

In this report, we present a case of cauda equina syndrome following postoperative spinal epidural hematoma, where urinary dysfunction was the primary presenting symptom, occurring without perineal sensory changes or lower extremity neurological deficits. (A) Postoperative lumbar MRI T2‐weighted axial revealed spinal epidural hematoma (red arrow) compressing the thecal sac and spinal canal stenosis at the surgical site. (B) The intraoperative image showed the consolidated hematoma.

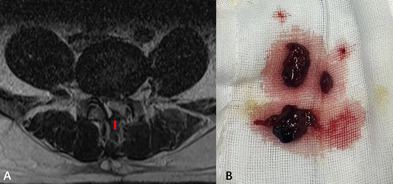

## 1 Introduction

Cauda equina syndrome (CES) is a rare but severe neurosurgical emergency primarily resulting from compression of the cauda equina nerve roots, most often caused by conditions such as central intervertebral disc herniation but also associated with trauma, hematomas, inflammatory conditions, postoperative complications, or tumors [1, 2]. CE Scan leads to irreversible neurological deficits, including lower limb paralysis and bowel and bladder dysfunction, if not promptly recognized and untreated. The annual incidence rate of cauda equina lesions is estimated at 3.4 per million population, with a period prevalence of 8.9 per 100,000 cases per year [2]. CES typically presents with a combination of bladder and/or bowel dysfunction, diminished sensation in the saddle area, and motor or sensory changes in the lower limbs, making it relatively straightforward to diagnose when these classic symptoms are present. Early recognition and prompt surgical decompression are critical to prevent permanent disability.

Postoperative spinal epidural hematoma (SEH) is a rare complication following lumbar laminectomy and decompression surgery, with an incidence rate around 0.1% [3]. SEH with neurological symptoms often requires emergent surgical evacuation. SEH may lead to CES by compressing the cauda equina, potentially resulting in devastating neurological outcomes [4, 5]. The early identification of SEH‐induced CES is crucial, but its diagnosis can be challenging when typical symptoms, such as lower limb weakness and sensory deficits, are absent. In particular, urinary retention—a common postoperative complaint after spinal anesthesia—may be overlooked or attributed to less serious causes, delaying critical intervention [6]. Early catheterization further complicates this complexity, which prevents bladder distension and overflow, potentially masking CES symptoms. In this case, we present a rare CES following lumbar microdiscectomy under spinal anesthesia, where postoperative SEH manifested exclusively as urinary retention without accompanying lower limb weakness or perineal sensory loss. This case underscores the importance of considering CES in postoperative patients presenting with isolated urinary symptoms, even without traditional neurological deficits. By discussing the diagnostic challenges and treatment outcomes in this case, we aim to contribute to the growing literature on atypical CES presentations and highlight the need for heightened vigilance in postoperative monitoring Table 1.

**TABLE 1:** Reported cases of cauda equina syndrome resulting from postoperative spinal epidural hematoma.

Author and publication date	Cases	Age/sex	Level	Surgical procedures	Symptom	Interval to the first surgery	Recovery after the second surgery	Recovery
Kaner et al. 2009 [3]	One	35/F	L5–S1	Discectomy	Leg weakness, hypoesthesia, and urine incontinence	12 h	4 months	Complete
Podnar 2010 [2]	Two	69/M	L5–S1	Spinal canal exploration	Severe pain, sensory symptoms, transient weakness, bladder, and bowel dysfunction	12 h	**—**	Incomplete
		73/F	L4–L5	Decompression	Severe pain, sensory symptoms, leg weakness, bladder, and bowel dysfunction	24 h	**—**	Incomplete
Kamoda et al. 2013 [9]	One	68/M	L2–L5	Laminectomies and fusion	Bladder retention	15 days	10 days	Complete
Present case	One	46/M	L4–L5	Discectomy	Painless bladder retention, overflow, and bowel dysfunction	8 h	3 months	Complete

### 1.1 Case Presentation

**FIGURE 1:**
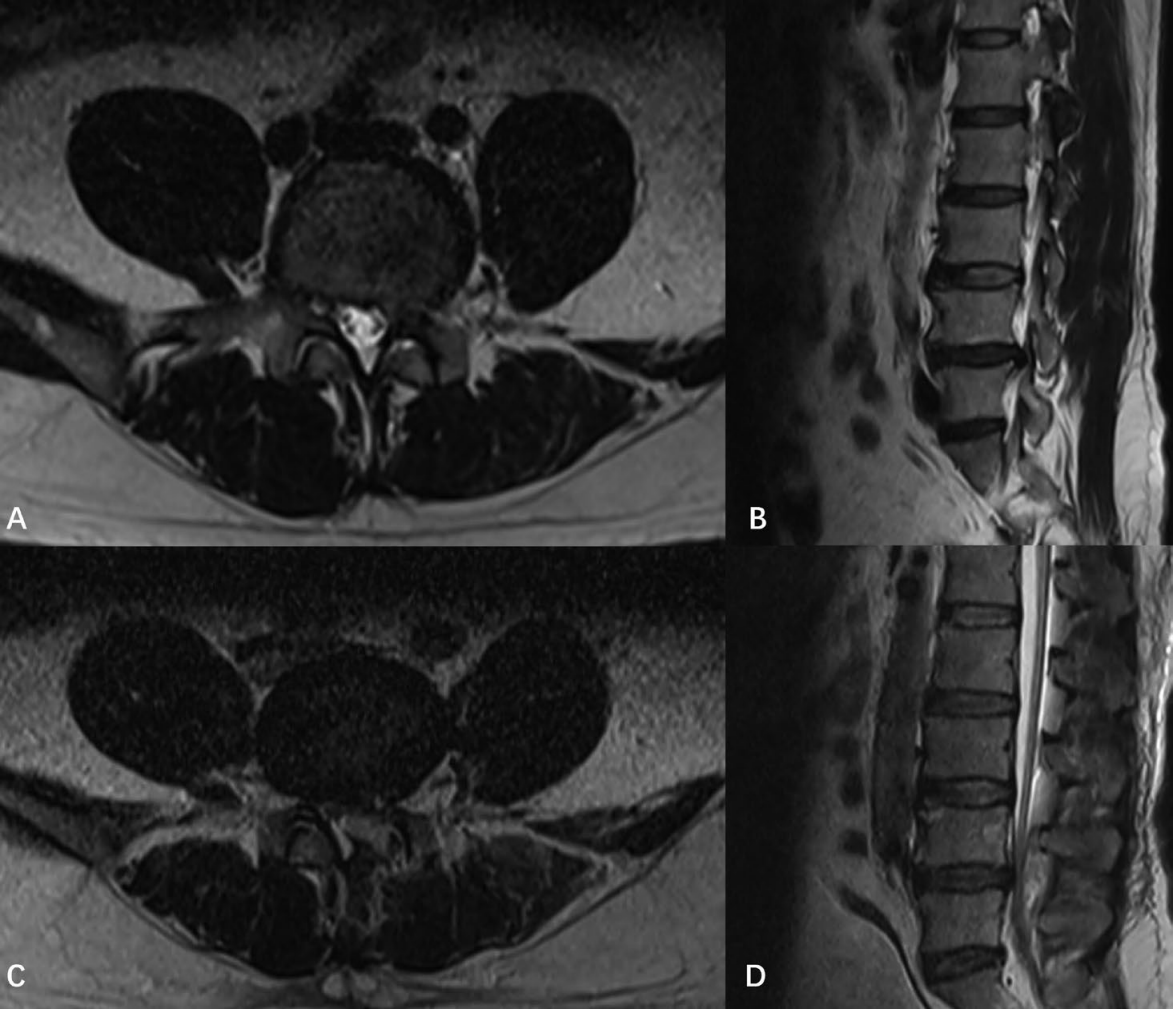
Preoperative MRI *T*_2_‐weighted axial (B) and sagittal (A) images show an L4‐L5 disc herniation with compression of the L5 nerve root. Postoperative MRI *T*_2_‐weighted axial (C) and sagittal (D) images reveal an SEH compressing the thecal sac and causing spinal canal stenosis at the surgical site.

A 46‐year‐old male presented with a one‐year history of left leg radiculopathy that was refractory to conservative management. The patient had a body mass index of 24.9 kg/m^2^ and no relevant medical history, including conditions such as hypertension or diabetes, associated with the current presentation. Preoperative magnetic resonance imaging (MRI) demonstrated L4–L5 disc herniation with compression of the left L5 nerve root (Figure 1a,b). The patient underwent an L4–L5 microdiscectomy under spinal anesthesia, with minimal intraoperative blood loss (approximately 20 mL), and no urinary catheterization. Following meticulous hemostasis at the surgical site, the wound was closed without the need for drainage.

Postoperatively, the patient experienced immediate relief from leg pain, and neurological function in the lower limbs remained intact. However, approximately 8 h after surgery, the patient developed urinary retention, necessitating the placement of a Foley catheter. On postoperative day 2, after catheter removal, the patient reported urinary frequency, urgency, and incomplete voiding, though no sensory or motor deficits were observed in the perineum and the leg. The patient had not passed a bowel movement and developed a middle‐grade fever, with a body temperature ranging between 38°C–38.5°C. On postoperative day 3, the patient experienced recurrent numbness and pain in the left lower limb, without accompanying muscle weakness or perineal sensory changes. Following the administration of steroids and analgesics, the numbness and pain in the left lower limb improved by postoperative day 4. However, urinary frequency, urgency, and incomplete voiding persisted. A urology consultation suggested that these symptoms were likely due to urethral irritation following the removal of the indwelling catheter. Tamsulosin hydrochloride sustained‐release capsules were prescribed to manage these urinary symptoms, but improvement was minimal.

By postoperative day 6, the patient experienced worsening urinary retention and developed fecal incontinence while straining to urinate. On postoperative day 7, these symptoms continued to escalate, although lower limb strength and perineal and leg sensations remained intact. Ultrasound revealed approximately 1500 mL of post‐void residual urine, indicating painless retention with overflow. MRI demonstrated SEH and spinal canal stenosis at the surgical site (Figure 1c,d).

**FIGURE 2:**
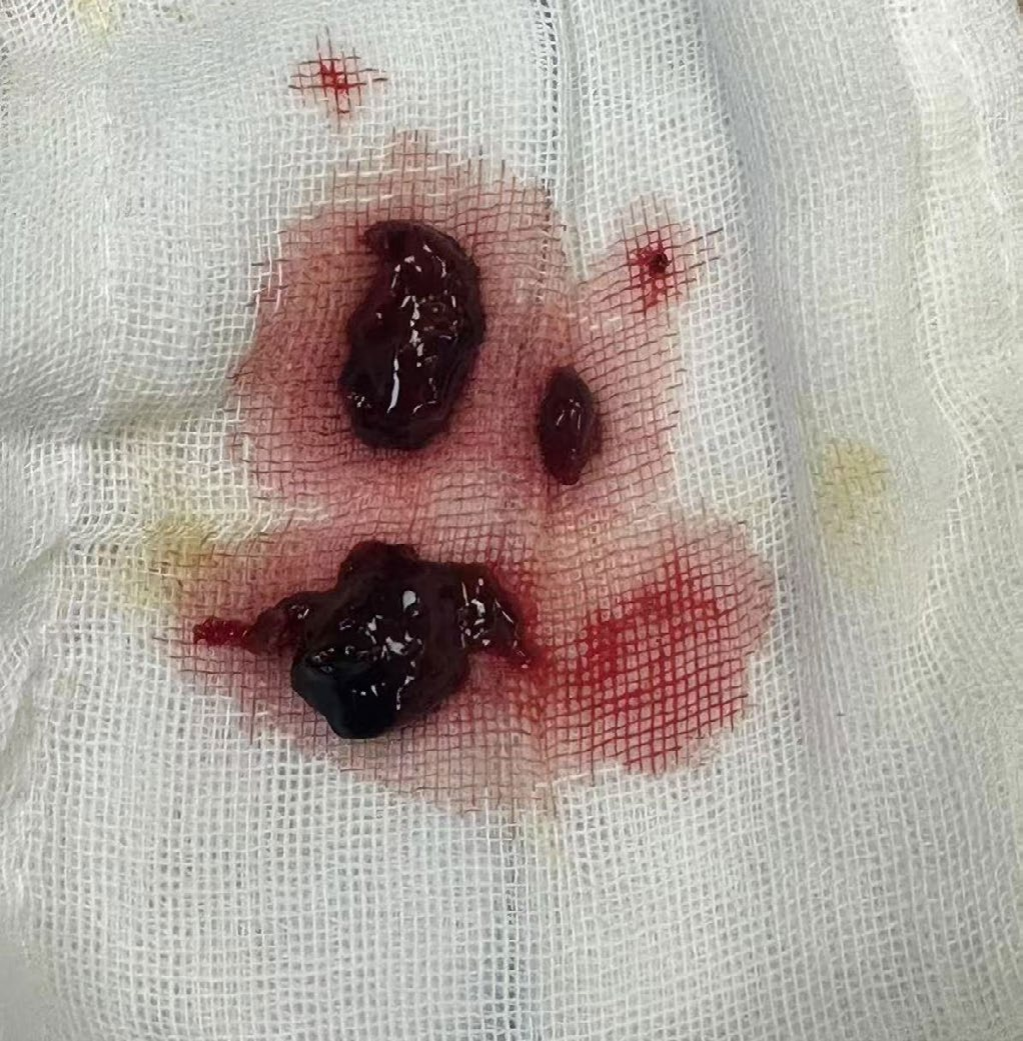
Intraoperative image depicting the consolidated hematoma.

A diagnosis of CES with painless bladder retention (CESR) due to postoperative SEH was made. The patient underwent emergency debridement and hematoma evacuation under general anesthesia (Figure 2), with a suction drain placed. By postoperative day 3, bowel function had normalized, and an erection was noted, but the patient remained unable to urinate spontaneously. He reported discomfort during gentle traction of the Foley catheter with the balloon inflated, indicating a preserved bladder sensation.

By 1.5 months postoperatively, the Foley catheter was no longer required. By 3 months, both bowel and bladder functions had fully recovered, allowing for spontaneous and complete voiding. Sexual function remained intact, and lower limb sensory and motor functions were fully intact.

## 2 Discussion

CES is a rare but serious neurological emergency following lumbar spine surgery, with an incidence of 0.08%–0.2% in patients undergoing lumbar surgery [7, 8]. It is typically characterized by bladder and/or bowel dysfunction, diminished sensation in the saddle area, sexual dysfunction, and potential neurological deficits in the lower limbs [1]. These hallmark symptoms are critical for early diagnosis and prompt surgical intervention, which is key to minimizing irreversible neurological damage. However, this case highlights a more unusual presentation of CES, where the patient's sole symptom was urinary dysfunction, and typical neurological deficits were notably absent, underscoring the clinical challenge in diagnosing CES when its presentation diverges from the classic symptomatology, particularly in the postoperative setting.

### 2.1 Literature Review of Postoperative SEH‐Induced CES

SEH is an uncommon but potentially devastating complication following lumbar spine surgery, with an incidence requiring surgical evacuation ranging from 0.1% to 0.2% [3]. In most cases, SEH‐induced CES presents with motor and sensory deficits in the lower limbs, facilitating early detection [2, 3]. Podnar reported two cases of postoperative CES caused by lumbar epidural hematoma after decompression surgery [2]. Both patients exhibited classical symptoms of sacral nerve involvement, including bladder, bowel, and sexual dysfunction, along with severe lower limb pain, sensory disturbances, and motor weakness. Early diagnosis was achieved due to these prominent neurological deficits, allowing emergency hematoma evacuation within 24 h. At follow‐up, both patients demonstrated significant improvement in lower extremity motor and sensory function. However, one patient regained normal bladder function but continued to experience involuntary flatus and inactive sexual function, while the other suffered persistent urinary and fecal incontinence with inactive sexual function [2].

Kaner et al. reported a case of CES secondary to lumbar epidural hematoma following L5‐S1 discectomy [3]. The patient developed neurological deficits, including urinary incontinence, lower extremity weakness, and hypoesthesia, within 12 h postoperatively. Prompt surgical evacuation of the hematoma and decompression led to complete recovery of motor function within 4 months. Notably, this case illustrates the possibility of atypical CES presentations, characterized by isolated urinary dysfunction without perineal sensory loss, lower limb weakness, or sensory deficits. These atypical manifestations underscore the importance of considering early urinary symptoms, including retention or urgency, as potential indicators of CES. Failure to recognize these signs could lead to delayed diagnosis and subsequent neurological deterioration. Supporting this, Kamoda et al. described a similar case of postoperative CES occurring 15 days after lumbar decompression and fixation surgery [9]. The patient presented with difficulty urinating but no leg or back pain. Emergency hematoma evacuation resulted in complete recovery of urinary function [9].

### 2.2 Urinary Retention as an Early Indicator of CES

In the postoperative setting, urinary retention is a common complaint, often attributed to temporary causes such as spinal anesthesia or opioid use. Typically, urinary retention due to spinal anesthesia resolves once the anesthesia's effects subside. Persistent urinary retention without improvement, however, should prompt clinicians to consider the possibility of CES. Painless urinary retention is a cardinal sign of the CES; in its absence, the likelihood of CES is estimated to be as low as 1 in 1000 [1, 4, 5]. However, in the absence of lower limb weakness or perineal sensory deficits, clinicians may not immediately suspect CES. The delay in diagnosing CES in this case was primarily due to the non‐specific nature of urinary dysfunction, which initially masked the severity of the underlying pathology. This case reinforces the necessity for heightened vigilance in monitoring postoperative patients for changes in urinary function, particularly when there is no apparent neurological cause. The findings align with Todd's recommendations that clinicians should remain alert to CES when micturition patterns change, even in the absence of classical symptoms like perineal sensory loss or lower limb motor deficits [10].

### 2.3 Timing of Surgical Intervention and Neurological Recovery

The timing of surgical intervention in CES is widely considered a critical factor in determining neurological outcomes, particularly bladder function. Several studies have shown that early decompression—preferably within 24 h—leads to significantly better outcomes in CES patients [3, 4, 5, 6]. Lavy et al. [1] recommended a two‐stage classification system, distinguishing between incomplete CES and CESR, to aid in prognostic evaluation. Incomplete CES is characterized by urinary dysfunction, such as altered voiding sensation, changes in the desire to urinate, or the need to strain, but with retained executive control of bladder function, allowing difficult but possible voiding. CESR, in contrast, reflects more severe neurological damage and carries a poorer prognosis, occurring when the bladder is no longer under voluntary control, leading to painless urinary retention with overflow [11]. Although some authors have suggested that once CESR has developed, the timing of surgery does not affect the outcome, emergency surgery is still necessary to prevent CESR from progressing to complete CES [1]. However, Dhatt et al. [12] found that patients undergoing earlier surgery for CES generally had better functional recovery, although some improvement can still be expected with aggressive decompression, even in CESR patients presenting later, and the outcomes are not universally poor as previously thought. In this case, although the surgery was delayed until postoperative day 7, the patient achieved a full neurological recovery. This could be attributed to several factors, including the absence of perineal sensation deficits and the preservation of bladder sensation by the S2/3/4 sensory supply, as indicated by the discomfort experienced during catheter traction. Dhatt et al. [12] have suggested that the absence of perineal numbness may serve as a positive prognostic indicator for bladder recovery, which is consistent with the outcome observed in this case.

### 2.4 Iatrogenic SEH and Risk Factors

While the exact etiology of SEH in this patient remains unclear, several risk factors for postoperative SEH have been identified in the literature, including inadequate intraoperative hemostasis, anticoagulant use, and lack of postoperative drainage [13, 14, 15]. Despite the absence of anticoagulation therapy and minimal intraoperative bleeding, the patient developed SEH, highlighting the unpredictable nature of this complication. Current evidence suggests that postoperative drains can reduce the incidence of SEH, particularly in multi‐segment decompression surgeries [14, 15]. In the present case, the absence of a drain may have contributed to the hematoma formation, though no definitive risk factors were identified. This underscores the need for further research into the optimal prevention strategies for SEH in high‐risk surgical patients.

### 2.5 Clinical Implications and Future Directions

This case provides valuable insights into the atypical presentation of CES following lumbar surgery. Clinicians should be aware that urinary dysfunction alone may be an early sign of CES, even in the absence of the classic motor and sensory deficits [16, 17]. This is particularly important in postoperative settings where early urinary symptoms may be dismissed as benign or transient. By recognizing these subtle early indicators, healthcare providers can intervene sooner, potentially improving patient outcomes.

Future studies should focus on developing more comprehensive guidelines for monitoring postoperative patients, especially regarding urinary function, to aid in the early detection of CES. Additionally, the role of postoperative drainage in preventing SEH, particularly in minimally invasive and multi‐segment surgeries, warrants further investigation to reduce the risk of this serious complication.

## 3 Conclusion

In summary, this case highlights the importance of recognizing CES in patients with isolated urinary dysfunction after lumbar surgery, even when typical neurological symptoms such as lower limb weakness or perineal sensory loss are absent. Early detection and prompt surgical intervention are critical, as delayed diagnosis may result in permanent neurological deficits. Moreover, this case underscores the need for vigilant postoperative monitoring, especially of urinary function, as isolated urinary symptoms may serve as early indicators of CES. Maintaining a high index of suspicion for CES, even in atypical cases, can facilitate timely diagnosis and improve patient outcomes.

Furthermore, this case highlights the necessity for continued research into the prevention of postoperative SEH and the development of more comprehensive diagnostic criteria for CES in postoperative patients. Future studies should prioritize creating robust guidelines for monitoring urinary function in postoperative patients to enable the early detection and treatment of CES.

## Author Contributions

The conceptualization was proposed by G.L. and B.Z. Literature collection and information analysis were performed by H.F.R. The first draft of the manuscript was written by G.L. and Q.L. The review and editing were performed by W.M. and Y.H.

## Ethics Statement

The study received approval from the Ethical Review Board of Ningbo No.6 Hospital (approval number: 202496L). Written informed consent for publication was obtained from the patient. This study only used the MRI images (without head region) of patients and information was anonymized. There is no concern about identifying information in the submission.

## Consent

We confirm that the work described has not been published before; that it is not under consideration for publication elsewhere; that its publication has been approved by all the authors and that its publication has been approved by the responsible authorities at the institution where the work was carried out. A statement regarding ethics approval and written informed consent was obtained from the patients for publication of this case report and accompanying materials.

## Conflicts of Interest

The authors declare no conflicts of interest.
